# Advanced Materials for Electrochemical Energy Storage: Lithium-Ion, Lithium-Sulfur, Lithium-Air and Sodium Batteries

**DOI:** 10.3390/ijms24033026

**Published:** 2023-02-03

**Authors:** Christian M. Julien

**Affiliations:** Institut de Minéralogie, de Physique des Matériaux et de Cosmologie (IMPMC), Sorbonne Université, UMR-CNRS 7590, 4 place Jussieu, 75005 Paris, France; christian.julien@sorbonne-universite.fr

The intention behind this Special Issue was to assemble high-quality works focusing on the latest advances in the development of various materials for rechargeable batteries, as well as to highlight the science and technology of devices that today are one of the most important and efficient types of energy storage, namely, lithium-ion, lithium–sulfur, lithium–air and sodium-ion batteries. High-performance battery technology is considered a key enabling factor for deep decarbonization via large-scale applications in electric vehicles. In addition, sustainable economic developments are possible by paying considerable attention to promoting sustainable and renewable energy sources. The exploitation of these intermittent types of energy systems requires adequate energy storage methods, wherein a significant role is played by batteries as versatile energy storage devices.

The contributions offer insight into a range of materials, the basic elements of batteries, with an approach enabling perspectives from the nano- to macroscopic. In these batteries, not only cathode and anode materials, but also other components, such as electrolytes, additives and separators, play crucial roles in determining their energy density, life-time, power capability, safety and cost. Special attention has been devoted to the design and synthesis of materials to achieve a stable electrochemical performance by introducing various functions derived from their special morphology and architecture, proper particle dimensions, surface engineering, doping and composite formation, etc. Therefore, the extensive study of battery materials plays an increasingly important role in producing advanced rechargeable batteries for the sustainable development of the future.

Elemental doping for substituting lithium or oxygen sites has become a simple and effective technique for improving the electrochemical performance of layered cathode materials. Compared with single-element doping, Wang et al. [1] presented an unprecedented contribution to the study of the effect of Na^+^/F^−^ cationic/anodic co-doping on the structure and electrochemical performance of LiNi_1/3_Mn_1/3_Co_1/3_O_2_. First principles calculations of 3D and 2D potential maps demonstrated that Na doping could reduce the potential well and increase the removal rate of Li^+^ ions [2]. The co-doped Li_1-z_Na_z_Ni_1/3_Mn_1/3_Co_1/3_O_2-z_F_z_ (*z* = 0.025) and pristine LiNi_1/3_Co_1/3_Mn_1/3_O_2_ materials were synthesized via the sol–gel method using ethylenediaminetetraacetic acid (EDTA) as the chelating agent. Structural analyses revealed that the Na^+^ and F^−^ dopants were successfully incorporated into the Li and O sites, respectively. The co-doping resulted in larger Li slab spacing, a lower degree of cation mixing and the stabilization of the surface structure, which substantially enhanced the cycling stability and rate capability of the cathode material. The Na/F co-doped electrode delivered an initial specific capacity of 142 mAh g^−1^ at a 1C rate (178 mAh g^−1^ at 0.1C), and it maintained 50% of its initial capacity after 1000 charge–discharge cycles at a 1C rate.

Bubulinca et al. [3] undertook a comparative study of binary and ternary self-standing composite cathode materials prepared using optimized binder-free technology. The binary “island–bridge” LiMn_2_O_4_/carbon nanotube (LMO/CNT) composite and the ternary “tectonic plate–island bridge” LiMn_2_O_4_/CNTs/graphene biomimetic structure were fabricated using a poly (ethylene glycol) p-isooctyl-phenyl ether (Triton X-100) as the surfactant. In the ternary composite, the graphene (Gr) substrates supported an entangled matrix of conductive CNTs, which connected the island of nonconductive inorganic materials composed of LMO. The typical spinel structure of LMO remains unchanged after modifying the basic structure with Gr and PANI due to a simplified hydrothermal method used for synthesis. The Gr and PANI core–shell coating improves the electric conductivity from 0.0025 up to 1 S cm^−1^. The electrochemical performance of the LMO/CNTs/Gr/PANI composite electrode can be optimized up to 136 mAh g^−1^ compared to 111 mAh g^−1^ for the LMO/CNTs. Besides that, the new electrode showed a good cycling stability after 200 galvanostatic charging/discharging cycles, making this structure a future candidate for cathode materials for aqueous rechargeable batteries.

In Abraham et al. [4], synthesis via the sol–gel technique and the electrochemical performance of a NASICON-based Na_3_V_2_(PO_4_)_2_F_3_ (NVPF) cathode material were reported on as an electrode of symmetric cells. It was noticed that the NVPF demonstrated a decent electrochemical behavior in a symmetric cell, exhibiting a reversible specific discharge capacity of ~85 mAh g^−1^ at a 0.1C rate and a cycling capacity retention of 61% after 1000 cycles at 1C. The salient characteristic of symmetric batteries is the use of the same cathode and anode materials, imparting number of advantages, such as (i) ease of fabrication, (ii) reduced costs (as only one type of material needs to be synthesized), (iii) enhanced electrochemical performance, (iv) the suppression of the volume expansion of the electrode materials, (v) reduced dendrite issues in LIBs and SIBs, (vi) improved safety and (vii) a longer battery life [5,6].

Hsu and coworkers [7] prepared multiporous carbons (MPCs) using ZnO as a hard template and biomass pyrolysis oil as the carbon source. It was shown that the surface area, pore volume and mesopore/micropore ratio of the as-prepared MPCs could be easily controlled by adjusting the ZnO/oil ratio. The sulfur/MPC (S/MPC) composite was prepared by blending sulfur powder with the as-prepared MPCs, followed by microwave heating at three different powers (100–300 W) for 60 s. The unique micro/mesostructure characteristics of the resulting porous carbons not only endowed the S/MPC composite with sufficient available space for sulfur storage, but also provided favorable and efficient channels for Li-ion/electron transportation. When applied as the electrode material in a lithium-ion battery, the S/MPC composite showed a reversible specific capacity of ~500 mAh g^−1^ and a high Coulombic efficiency (>95%) after 70 cycles. Overall, the method proposed in this study provides a simple and green approach for the rapid production of MPCs and S/MPC composites for high-performance batteries.

To overcome the technological problems encountered in Li–S batteries, i.e., the shuttle effect, electronic insulation of S_8_ and Li_2_S and high fluctuation of electrode volume after multiple cycles, MXenes, which are a type of emerging two-dimensional materials, have been successfully used. Tian et al. [8] reviewed the recent advances in MXene-based materials as cathodes for Li–S batteries. Several synthetic strategies were summarized alongside a discussion of the excellent electrochemical properties of MXenes.
